# Comparison of: (2*S*,4*R*)-4-[^18^F]Fluoroglutamine, [^11^C]Methionine, and 2-Deoxy-2-[^18^F]Fluoro-*D*-Glucose and Two Small-Animal PET/CT Systems Imaging Rat Gliomas

**DOI:** 10.3389/fonc.2021.730358

**Published:** 2021-10-07

**Authors:** Maxwell W. G. Miner, Heidi Liljenbäck, Jenni Virta, Semi Helin, Olli Eskola, Petri Elo, Jarmo Teuho, Kerttu Seppälä, Vesa Oikonen, Guangli Yang, Andrea Kindler-Röhrborn, Heikki Minn, Xiang-Guo Li, Anne Roivainen

**Affiliations:** ^1^ Turku PET Centre, University of Turku, Turku, Finland; ^2^ Turku Center for Disease Modeling, University of Turku, Turku, Finland; ^3^ Turku PET Centre, Turku University Hospital, Turku, Finland; ^4^ Organic Synthesis Core Facility, Memorial Sloan Kettering Cancer Center, New York, NY, United States; ^5^ Institute of Pathology, University Hospital of Essen, University of Duisburg-Essen, Essen, Germany; ^6^ Department of Oncology and Radiotherapy, Turku University Hospital, Turku, Finland; ^7^ InFLAMES Research Flagship Center, University of Turku, Turku, Finland

**Keywords:** fluoroglutamine, methionine, FDG, glioma, modeling, rat, positron emission tomography, PET

## Abstract

**Purpose:**

The three positron emission tomography (PET) imaging compounds: (2*S*,4*R*)-4-[^18^F]Fluoroglutamine ([^18^F]FGln), *L*-[methyl-^11^C]Methionine ([^11^C]Met), and 2-deoxy-2-[^18^F]fluoro-*D*-glucose ([^18^F]FDG) were investigated to contrast their ability to image orthotopic BT4C gliomas in BDIX rats. Two separate small animal imaging systems were compared for their tumor detection potential. Dynamic acquisition of [^18^F]FGln was evaluated with multiple pharmacokinetic models for future quantitative comparison.

**Procedures:**

Up to four imaging studies were performed on each orthotopically grafted BT4C glioma-bearing BDIX rat subject (n = 16) on four consecutive days. First, a DOTAREM^®^ contrast enhanced MRI followed by attenuation correction CT and dynamic PET imaging with each radiopharmaceutical (20 min [^11^C]Met, 60 min [^18^F]FDG, and 60 min [^18^F]FGln with either the Molecubes PET/CT (n = 5) or Inveon PET/CT cameras (n = 11). *Ex vivo* brain autoradiography was completed for each radiopharmaceutical and [^18^F]FGln pharmacokinetics were studied by injecting 40 MBq into healthy BDIX rats (n = 10) and collecting blood samples between 5 and 60 min. Erythrocyte uptake, plasma protein binding and plasma parent-fraction were combined to estimate the total blood bioavailability of [^18^F]FGln over time. The corrected PET-image blood data was then applied to multiple pharmacokinetic models.

**Results:**

Average BT4C tumor-to-healthy brain tissue uptake ratios (TBR) for PET images reached maxima of: [^18^F]FGln TBR: 1.99 ± 0.19 (n = 13), [^18^F]FDG TBR: 1.41 ± 0.11 (n = 6), and [^11^C]Met TBR: 1.08 ± 0.08, (n = 12) for the dynamic PET images. Pharmacokinetic modeling in dynamic [^18^F]FGln studies suggested both reversible and irreversible uptake play a similar role. Imaging with Inveon and Molecubes yielded similar end-result ratios with insignificant differences (p > 0.25).

**Conclusions:**

In orthotopic BT4C gliomas, [^18^F]FGln may offer improved imaging *versus* [^11^C]Met and [^18^F]FDG. No significant difference in normalized end-result data was found between the Inveon and Molecubes camera systems. Kinetic modelling of [^18^F]FGln uptake suggests that both reversible and irreversible uptake play an important role in BDIX rat pharmacokinetics.

## Introduction

With an increasing number techniques and compounds available in neuro-oncology, it becomes important to contrast and compare their abilities to image and diagnose the tumors. While magnetic resonance imaging (MRI) provides the highest *in vivo* spatial resolution, it still lacks the ability to detect specific cellular changes in metabolic activity. Positron emission tomography (PET) imaging has been used to successfully image regional metabolic activity since the development and use of the [^18^F]fluorine-labelled glucose analog 2-deoxy-2-[^18^F]fluoro-*D*-glucose ([^18^F]FDG) (**Figure 1.1**) in 1976 (1). Although [^18^F]FDG is still the most widely used radiopharmaceutical for PET imaging today (2), the search for and development of new and improved imaging radiopharmaceuticals continues. Considering the genetic differences displayed by various cancers, personalized radiopharmaceuticals for imaging specific *loci*, types and strains of tumors offers a promising strategy for personalized care.

**Figure 1:**
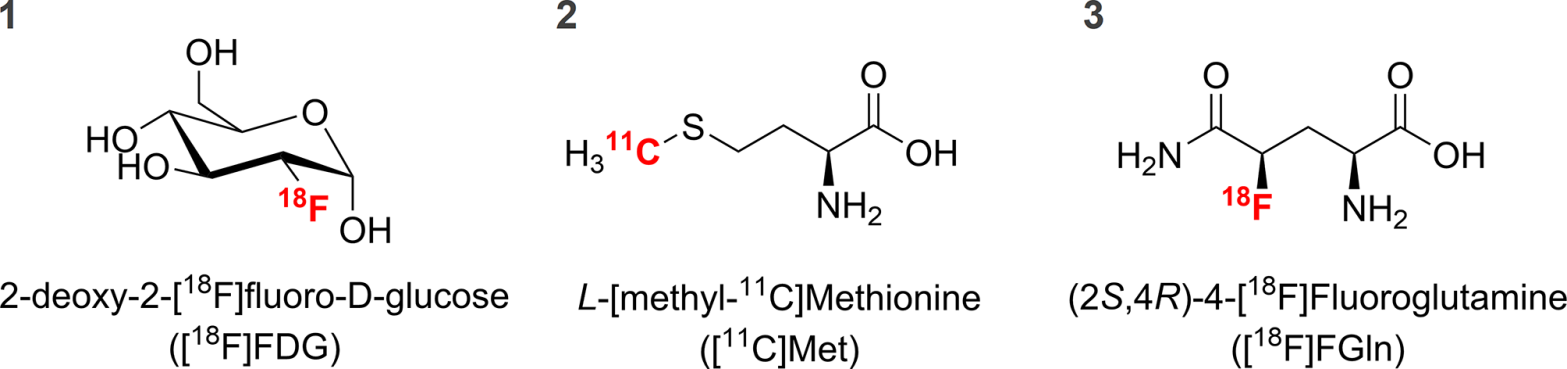
Chemical structure of the PET radiopharmaceuticals used in this study.

Radionuclide-labelled amino acids and amino-acid analogues are radiopharmaceuticals of choice in the PET imaging of gliomas because of their low uptake in normal brain tissue. These compounds are generally well transported throughout the body due to biochemical ubiquity and can have an increased uptake in metabolically-active or dividing tissues such as tumors (3). Amino acids outperform [^18^F]FDG because the cerebral cortex relies heavily on high glucose metabolism thus reducing contrast between the lesion and normal brain (4). Some amino acid radiolabeled analogues can divulge insight into irregular amino acid accumulation, which is often a trait present in tumors due to increased protein synthesis rates (5). An important and widely used amino acid radiopharmaceutical for brain tumor imaging is *L*-[methyl-^11^C]methionine ([^11^C]Met) (**Figure 1.2**) (6), which has seen use in humans for nearly the same time as [^18^F]FDG (7). Methionine in humans is an essential amino acid, which needs to be ingested from external sources and then transported throughout the body. It is the amino acid encoded by the start codon for most proteins and therefore its use and subsequent cellular pool depletion is tied heavily to the commencement of protein synthesis. Although it can be present in specific peptide chains in high amounts (8, 9), its average frequency of occurrence is lower than the majority of other amino acids (10, 11). Due to this, methionine cellular pool depletion and subsequent increased [^11^C]Met cellular uptake rates by cancer cells can be inherently variable depending on mutational status.

One of the more recently developed radiolabeled amino acid analogues is (2*S*,4*R*)-4-[^18^F]fluoroglutamine ([^18^F]FGln) (**Figure 1.3**), which has many similar properties to native glutamine *in vivo*. It is widely transported across the body and used for both protein synthesis and energy production (12). These dual complementary pathways, which deplete the cellular glutamine pool, can cause a substantial equilibrium shift in cancer cells leading to increased import, especially if mutations favor increased glutamine use for energy production. The relationship between increased glutamine consumption and cancer cells has long been studied with some cancers even being given the label of "glutamine addicted" (13). Considering the low level of basal glutamine uptake in the brain, [^18^F]FGln may be able to better differentiate between healthy and diseased cancerous tissue. Early results from the ongoing clinical trials in the USA (National Library of Medicine ID: NCT01697930) and other human-tested reports published thus far (14–16) suggest a promising future outlook for the radiopharmaceutical. It remains to be seen, however, if [^18^F]FGln will see future routine clinical use. Despite reports of *in vivo* metabolism instability (15), its usefulness for brain imaging may still be beneficial if ultimately improving tumor detection and patient outcomes.

To test new radiopharmaceuticals and disease models in an *in-vivo* preclinical setting, specially designed small-animal PET/computed tomography (CT) camera systems are usually employed. Testing of PET cameras is generally accomplished by using "phantom subjects" which are multi-chambered vessels of known dimensions that can be filled with known amounts of radioactivity. Camera testing usually adheres to standardized guidelines such as the National Electrical Manufacturers Association (NEMA) NU4-2008 (17). Still, there is some benefit in testing PET camera systems with *in vivo* subjects, but not all research centers have multiple camera systems and the variability in animal subjects complicates things further. Since its release, roughly 16 years ago, the Inveon Multimodality PET/CT (Siemens, Knoxville, TN, USA) has been a popular camera system for preclinical radiopharmaceutical research. The device has undergone improvements to its image processing capabilities and could be considered the standard camera for preclinical PET imaging. A more recently established company, Molecubes NV (Gent, Belgium), has released a modular bench-top camera system for CT, PET and single-photon emission computed tomography (SPECT). These modular cubes take up a small amount of lab space and are (comparably) easily transportable making it difficult to imagine such a device can deliver similar quality to a more expensive and larger system. While it remains to be seen if the smaller modular camera systems will begin to establish themselves as a standard instrument, initial reports of system quality are promising (18, 19).

In this article we present our continued success in synthesizing [^18^F]FGln and our comparative *in vivo* PET imaging evaluation thereof juxtaposed with the well-established radiopharmaceuticals [^18^F]FDG and [^11^C]Met to image BDIX rats bearing BT4C gliomas. We tested two separate camera systems by conducting and analyzing parallel subject groups to compare the cameras' abilities. Finally, we have also included results from four separate pharmacokinetic model evaluations of [^18^F]FGln to ensure a plethora of quantitative data for future reference and comparison with newly developed radiopharmaceuticals.

## Methods

### Animal Models

The 16 glioma-bearing BDIX-Ifz rats (herein "BDIX") for imaging studies (8 weeks old, 9 males and 7 females) were anesthetized under 2-2.5% isoflurane and given buprenorphine (0.01 mg/kg) and carprofen (5 mg/kg) for analgesia during surgery. Subjects were injected with BT4C rat glioma cells (10,000 cells in 5 µL of plain growth medium) into the right hemisphere of the brain after drilling a small hole in the skull (location: 1 mm posterior bregma, 2 mm lateral right). The rats were provided food and water *ad libitum* and monitored for 3 weeks to allow optimal tumor growth before the (up to) 4 day imaging protocol was commenced. The metabolism study used 10 healthy BDIX rats (5 male and 5 female) and all animals in the study were provided food and water *ad libitum*. All animals were sacrificed after either the final imaging (PET studies) or the final blood sample collection (metabolism studies) under deep isoflurane anesthesia (4%) *via* cervical dislocation after cardiac puncture to collect terminal blood samples. The National Animal Experiment Board of Finland and the Regional State Administrative Agency for Southern Finland approved all animal work (permission no. ESAVI/12691/04.10.07/2017) and all animal handling was carried out under compliance with the European Union directive regarding the conduct of animal experimentation.

### Radiochemical Preparation of [^18^F]FGln, [^18^F]FDG, and [^11^C]Met

[^18^F]FDG (**Figure 1.1**) was prepared using standard FASTLab^®^ cassettes (20) and [^11^C]Met (**Figure 1.2**) with well-established synthesis protocols (see **Supplementary Material** for complete details). [^18^F]FGln (**Figure 1.3**) was prepared similarly to (21) with synthesis modifications previously listed in (22). All radiopharmaceuticals were analyzed for radiochemical purity with established high-performance liquid chromatography methods.

### *In Vivo* Imaging

The imaging was planned so that all subjects (n = 16) would receive a Dotarem^®^ (Guerbet LLC, Villepinte, France) contrast-enhanced MRI (Achieva 3T MRI, Koninklijke Philips N.V., Eindhoven, Netherlands) with a rat head specific coil (Rat brain array coil 4, RAPID Biomedical GmbH, Rimpar, Germany) on the first day followed by up to 3 PET/CT scans on the 3 following days. Imaging radiopharmaceutical order was random. All imaging was done while subjects were under 2% isoflurane maintenance anesthesia and allowed to recover immediately after if terminal studies were not forthcoming. Five subjects were imaged with the Molecubes X-Cube and β-Cube (Molecubes NV, Gent, Belgium) and eleven subjects were imaged with the Inveon Multimodality PET/CT scanner (Siemens Medical Solutions, Knoxville, TN, USA) (**Table 1**). Some subjects were sacrificed for *ex vivo* studies after PET imaging for each radiopharmaceutical. Of the 16 imaged subjects, 4 received PET/CT imaging from one radiopharmaceutical, 8 were imaged with two, and 4 were imaged with all three compounds.

**Table 1:** Radiopharmaceuticals, imaging systems, and PET/CT image amounts.

Camera system	Radiopharmaceutical			Total number of images (n)	Total number of subjects (n)
	[^18^F]FGln (n)	[^11^C]Met (n)	[^18^F]FDG (n)		
**Inveon**	9	8	4	21	11
**Molecubes**	4	4	3	11	5
**Total**	13	12	7	32	16

Dynamic PET image duration, framing, varied by radiopharmaceutical and were as follows: [^18^F]FGln - 6 × 10 s, 4 × 60 s, 5 × 300 s, 3 × 600 s, [^11^C]Met - 6 × 10 s, 8 × 30 s, 5 × 60 s, 2 × 300 s, and [^18^F]FDG - 6 × 10 s, 4 × 60 s, 5 × 300 s, 3 × 600 s. The injected amount of radioactivity also varied based on camera and radionuclide. For the Molecubes camera, approximately 5 MBq of radioactivity was injected for [^18^F]FDG and [^18^F]FGln, and 10 MBq for [^11^C]Met. For the Inveon camera, approximately: 50 MBq of [^11^C]Met, 10 MBq of [^18^F]FDG, or 15 MBq of [^18^F]FGln radioactivity was injected. All PET data was back corrected for radionuclide decay and acquired sinograms were reconstructed with the ordered subset expectation maximization 3-dimensional (OSEM-3D) algorithm.

### *In Vivo* Imaging Analysis

All PET/CT image analyses were performed with Carimas software (available for free at: http://turkupetcentre.fi/carimas/Turku PET Center, Finland). The (up to) three separate PET/CT images of the same subject with each imaging radiopharmaceutical were aligned with the corresponding contrast-enhanced T1-weighted MRI image. Each PET image was separately examined, in the initial 20 s of frames for the amino-acid-based radiopharmaceuticals and 40-60 min frames for [^18^F]FDG, to manually delineate heart left ventricle cavity voxels to use as a blood radioactivity estimation region of interest (ROI). Each frame was examined to ensure a lack of subject movement and corrected for by additional analysis post-movement on very rare occasion. Tumor and whole-brain boundaries were drawn using the contrast-enhanced T2-weighted MRI image as a reference through each transaxial slice (**Supplementary Figure 1A**) before being converted into 3D space (**Supplementary Figures 1B, C**). A second "expanded tumor" ROI was made by expanding the tumor volume in all directions (not shown) with the expanded volume subtracted from the whole brain volume to establish all "healthy brain" tissue excluding a roughly 2-voxel margin between boundaries in an unbiased fashion (**Supplementary Figure 1D**).

The dynamic PET image data was then extracted from each separate image and converted to standardized uptake values (SUV) which account for ROI volume, mean ROI radioactivity, subject weight, and total injected radioactivity dose (corrected for decay, residual syringe and cannula radioactivity). The results are shown as SUV ROI radioactivity curves over time as well as tumor-to-healthy brain region (TBR) ratios for improved comparisons. [^18^F]FGln uptake data and blood radioactivity data (corrected for bioavailability fraction over time) were also input into multiple pharmacokinetic models described in the *"[^18^F]FGln Pharmacokinetic Modeling"* section.

In an attempt to unbiasedly display visual differences in PET camera-specific data with a large difference in injected radioactivity, the following parameters were observed: The visual interpolation method was chosen to be tri-cubic to highlight noise with some additional nearest-neighbor interpolations to illustrate voxel size differences. Different radiopharmaceutical-images relevant to this study were chosen with additional images of other common tissues-of-interest included in the **Supplementary Material** (**Supplementary Figures 2–4**).

### [^18^F]FGln *In Vivo* Stability Analysis

Additional healthy BDIX rats (n = 10, 5 male, 5 female) were injected with approximately 40 MBq of [^18^F]FGln and 150–200 µL aliquots of blood drawn at 5, 15, 30, 45, and 60 min into a heparin coated vial at 5°C to be assayed. During the final 60 min collection time point, animals were sacrificed under deep anesthesia via cardiac puncture and cervical dislocation. All radioactivity measurements were performed with a 3″ Nal system (Triathler, Hidex Oy, Turku Finland). Whole blood was weighed and measured for radioactivity before being centrifuged at 700 ×*g* and 4°C for 5 min. The plasma was separated out before being weighed and gamma counted followed by storage on ice for the later described assays. Hematocrit values used were averaged separately for male and female populations with five samples for each group. The following Equation 1 (23) was then used to calculate the radioactivity fraction taken up by the red blood cells and the available plasma fraction to avoid including plasma contamination in the blood cell fraction:


Equation 1 $$Plasma\;Radioctivity\;Fraction=\frac{C_{p}}{C_{RBC}+C_{p}}=\frac{\rho_{p}C_{p}(HCT)}{\rho_{b}C_{b}-\rho_{p}C_{p}(1-HCT)+\rho_{p}C_{p}(HCT)}$$

Where *C* = radioactivity concentration, *ρ* = density, *RBC* = red blood cell, and HCT = hematocrit. Subscripts denote the following: *p* = plasma, *RBC* = red blood cell, and b = whole blood.

Plasma aliquots (80 µL) were then precipitated with methanol (200 µL) before being vortexed thoroughly and centrifuged at 11,000 ×*g* for 10 min. The two fractions were separated and measured for radioactivity to calculate the unbound radioactivity fraction of the plasma.

Aliquots of precipitated plasma supernatant were then analyzed to divulge the parent-radiopharmaceutical purity via established high-performance liquid chromatography methods (24) further described in the **Supplementary Material** with sample chromatogram **Supplementary Figure 5**.

The three values, total plasma radioactivity fraction, unbound plasma-activity fraction, and unbound plasma radioactivity fraction parent-radiopharmaceutical purity were multiplied for each sample (**Supplementary Figures 6A–C**) to produce **Figure 6**. The resulting Equation 2 approximates the bioavailable fraction of [^18^F]FGln from the total whole blood radioactivity at post-injection times in minutes to be used as a method of correcting blood inputs in the modeling methods.

### *Ex Vivo* Studies

*Ex vivo* biodistribution studies were performed for [^18^F]FGln with a complete organ list and results are listed in the **Supplementary Material** Some subjects from each radiopharmaceutical imaging group were sacrificed for autoradiographical and histological studies immediately after PET imaging. All autoradiography images were analyzed with Carimas software (Turku PET Centre, Turku, Finland).

### Statistical Methods and [^18^F]FGln Modeling

Blood input values from the heart left ventricle ROI in the PET image data were corrected for bioavailable fraction with Eqn. 2. Logan (25), Patlak (26), and two two-compartment models (27) (reversible and irreversible) were applied to whole-blood, unbound plasma fraction, and unbound plasma fraction assayed for parent-radiopharmaceutical purity as inputs for tumor and healthy brain ROIs. Mean, standard deviation, and two-tailed unpaired Student's *t*-test (for camera comparison, modeling values, and gender differences) p-values are reported where applicable.

## Results

### *In Vivo* Imaging and Analyses

**Figure 2:**
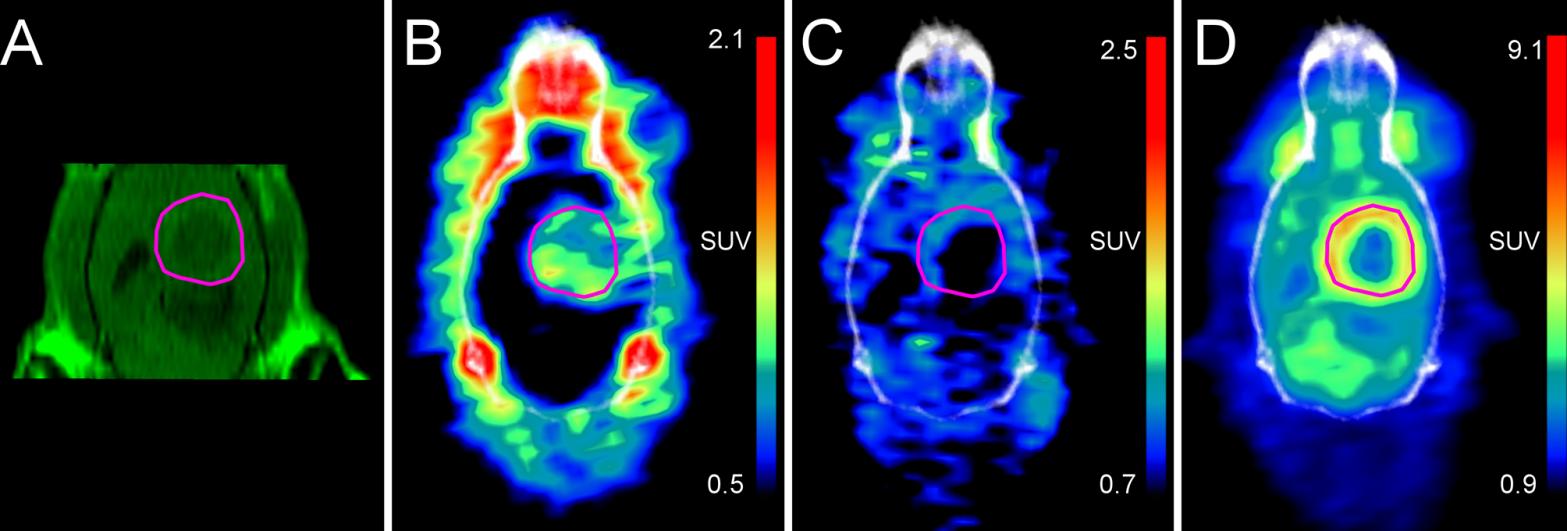
Comparative *in vivo* coronal-image array of a single BDIX rat subject's head region with MRI-based boundaries of a BT4C glioma (outlined in magenta) imaged on four consecutive days (PET/CT images depicted are from the Inveon PET/CT camera system). **(A)** MRI, **(B)** PET/CT, 15.8 MBq injection of [^18^F]FGln, time-weighted mean of frames 15-60 min **(C)** PET/CT, 50.4 MBq injection of [^11^C]Met time-weighted mean of frames 9-20 min **(D)** PET/CT, 24.0 MBq injection of [^18^F]FDG, time-weighted mean of frames 35-60 min.

**Figure 3:**
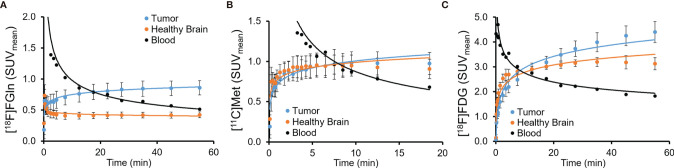
Average PET image time-radioactivity curves of all subjects' PET/CT images **(A)** [^18^F]FGln (n = 13), **(B)** [^11^C]Met (n = 12), **(C)** [^18^F]FDG (n = 7). Standard deviation error bars on blood radioactivity curves were omitted for clarity (shown in **Supplementary Figure 7**).

**Figure 4:**
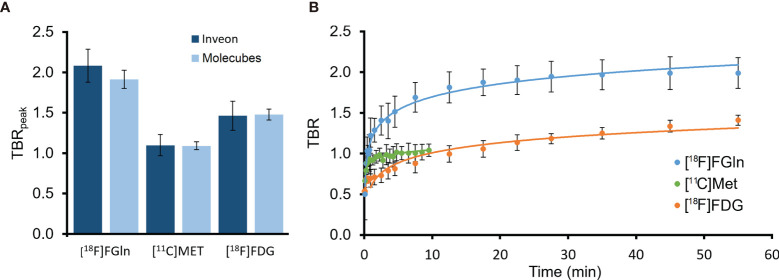
**(A)** Average peak-TBR levels reached during PET imaging for each radiopharmaceutical, separated by PET camera used Inveon [^18^F]FGln (n = 9), Molecubes [^18^F]FGln (n = 4), Inveon [^11^C]Met (n = 8), Molecubes [^11^C]Met (n = 4), Inveon [^18^F]FDG (n = 4), and Molecubes [^18^F]FDG (n = 3). **(B)** Both-camera average PET image radiopharmaceutical uptake tumor-to-healthy-brain tissue ratio (TBR) curves for BT4C glioma-bearing BDIX rats for [^18^F]FGln (n = 13), [^11^C]Met (n = 12) and [^18^F]FDG (n = 7).

**Figure 5:**
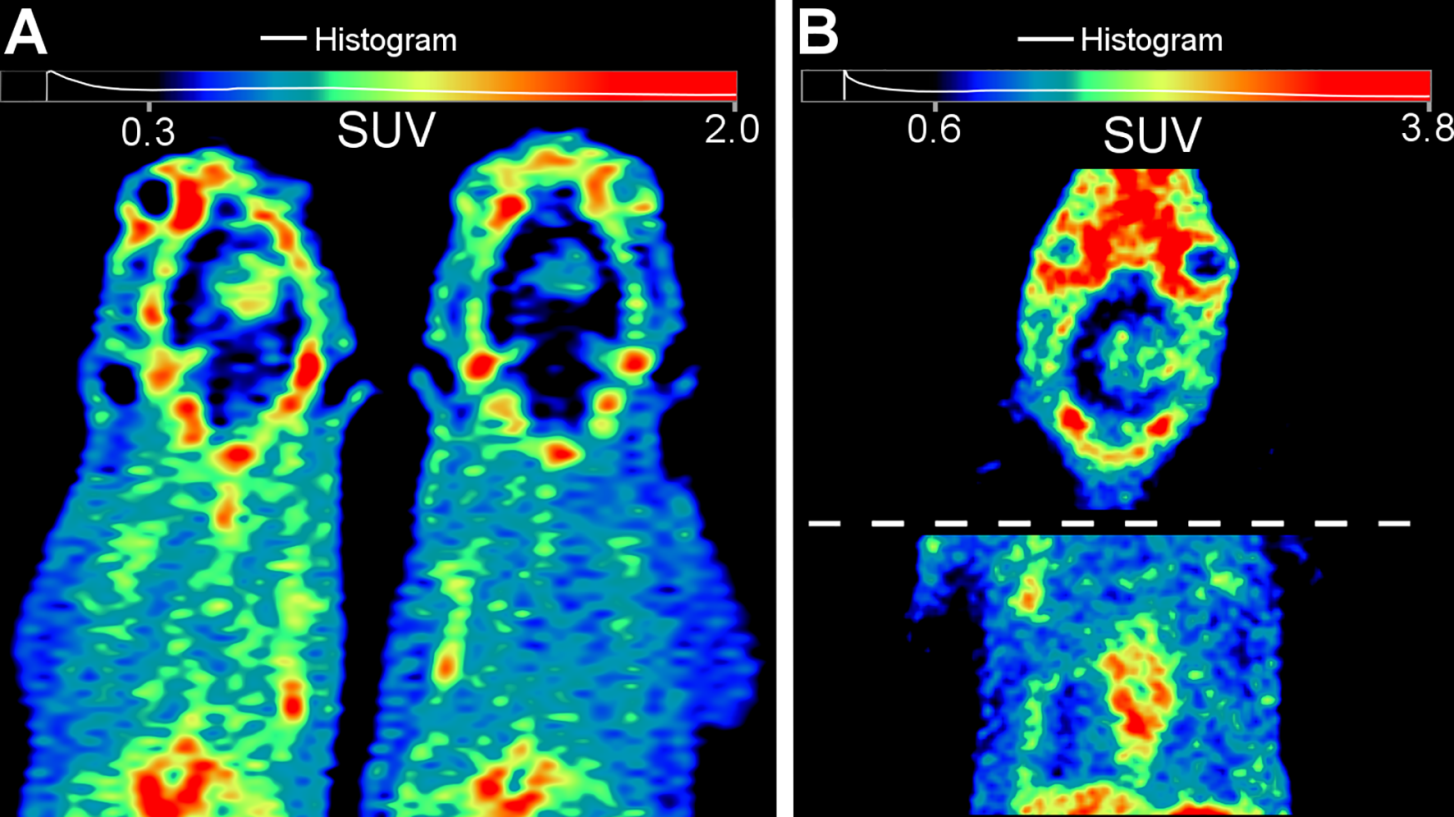
Sample visual camera-comparison of coronal [^18^F]FGln PET image slices with tri-cubic interpolation of time-weighted mean frames from 4 – 40 min post-injection. **(A)** Inveon PET/CT reconstructed with manufacturer recommendations (2 OSEM3D and 18 MAP iterations) with both subjects injected with approximately 15.2 MBq each. **(B)** Molecubes camera system (reconstructed with manufacturer recommendations (30 OSEM3D iterations) with the subject injected with 4.2 MBq. *note: To show similar body areas, the head and torso were split in **(B)** due to body positioning and gantry differences between each camera not showing the same region in a single coronal-plane slice. Additional organ and image comparisons presented in the **Supplementary Materials** Results section.

MRI-guided tumor delineation in PET images (sample single-subject **Figure 2**) yielded average time-activity curves (TACs) for all 32 PET/CT scans in **Figure 3** (blood TACs with error bars in **Supplementary Figure 7**). There was no significant difference (p > 0.25) in tumor-to-healthy brain ratios for both camera systems used in this study (**Figure 4A**) and thus data was pooled together. The differentiation between tumor and background brain uptake for each radiopharmaceutical appears most visually for [^18^F]FGln in **Figure 2** and was confirmed statistically via average tumor-to-background ratio curves shown in **Figure 4B** which reach their maxima of: 1.99 ± 0.19 (n = 13) for [^18^F]FGln, 1.08 ± 0.08 (n = 12) for [^11^C]Met, and 1.41 ± 0.11 (n = 7) for [^18^F]FDG. A sample wide field [^18^F]FGln PET image (**Figure 5**) for both camera systems was included (with additional in the **Supplementary Material** of the kidney, heart, and brain) to illustrate *in-vivo* differences in noise profiles and voxel size (Molecubes: 0.4 × 0.4 × 0.4 mm and Inveon 0.78 × 0.78 × 0.80 mm).

### *In Vivo* [^18^F]FGln Stability

**Figure 6:**
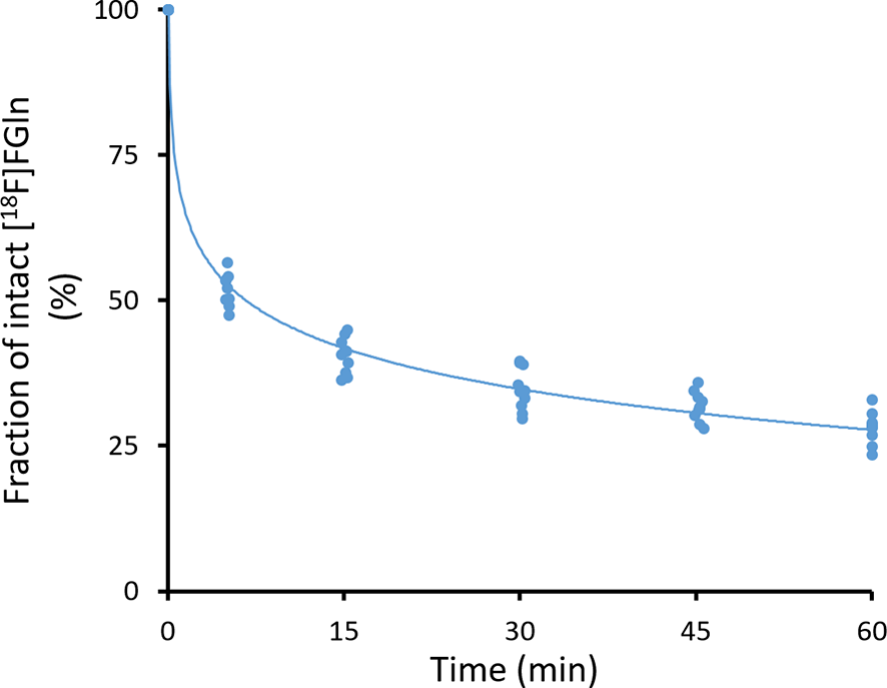
Total [^18^F]FGln bioavailability fraction of whole blood radioactivity curve for healthy male and female 3-month-old BDIX rats.

The individually assayed components of blood stability (ESM **Supplementary Figures 6A–C**) were multiplied to estimate the total parent-radiopharmaceutical bioavailable fraction of whole blood radioactivity (**Figure 6**). The healthy 3-month-old BDIX rats (n = 10) showed no significant (p > 0.9) difference between male (n = 5) and female (n = 5) populations with regard to overall nor individual components measured and were thus combined into one group. Parent-radiopharmaceutical stability assays revealed the total bioavailable fraction of [^18^F]FGln of the whole-blood radioactivity could be estimated by the well-fitting Equation 2. After approximately 60 min post-injection, the average (n = 10) bioavailable [^18^F]FGln-fraction of total blood radioactivity dropped to 28.1% ± 2.7%.


Equation 2 $${[}^{18}F]FGln\;Intact\;Fraction\;(t)=(I_{p})(-0.101)\ln(t)+0.692\;R^{2}=0.9887$$

Where *t* is the post-injection time in minutes and *I_p_* is the initial radiochemical purity injected

### [^18^F]FGln Modeling

**Figure 7:**
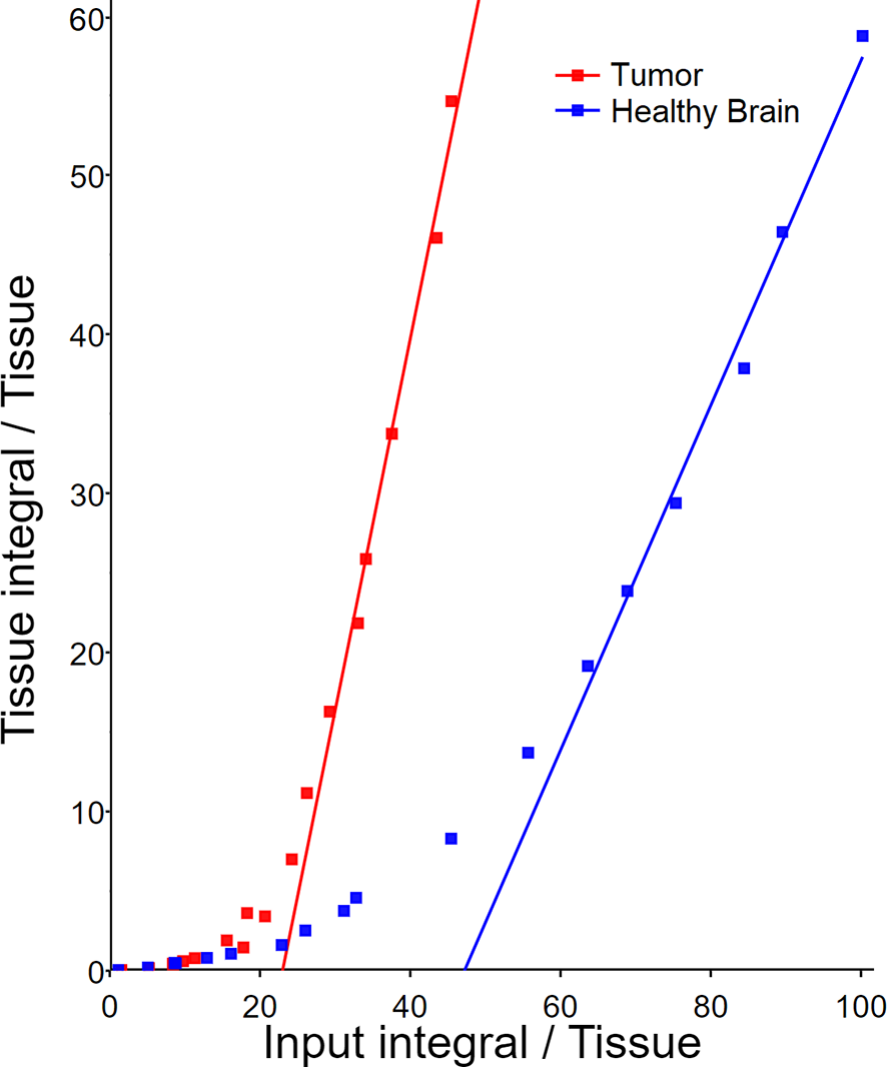
Sample Logan plot of dynamic [^18^F]FGln PET image tissue uptake with population-based metabolite-corrected bioavailable (free parent) plasma fraction of arterial blood as the input.

Mean radioactivity values over time for glioma, healthy brain, and blood or plasma (including and excluding corrections for bioavailable plasma fraction consisting of [^18^F]FGln with Eqn. 2) ROIs were input into Patlak, Logan, reversible and irreversible, 2-compartment models. The Logan plot (sample in **Figure 7**) revealed (free-parent plasma purity inputs) average (n = 13) distribution volumes of 4.00 ± 1.96 and 1.72 ± 0.66 for glioma and healthy brain ROIs respectively (**Table 2**). When examining Patlak plots (data in ESM) in comparison to Logan plots, the data suggested that in BDIX rats both irreversible and reversible uptake play a role in pharmacokinetics with plots showing similar degrees of linearity and magnitude. The complete modeling result tables along with sample plots are shown in the ESM (**Supplementary Tables 1–4 **and** Supplementary Figures 8–10**).

**Table 2:** Logan plot [^18^F]FGln pharmacokinetic (reversible uptake model) result summary.

Region of interest (n = 13)	DV (AB)	DV (P)	DV (FP)	Tumor DV/Healthy Brain DV (FP)
Tumor	1.35 ± 0.36	1.00 ± 0.30	4.00 ± 1.96	2.31 ± 0.40
Healthy brain	0.66 ± 0.19	0.48 ± 0.14	1.72 ± 0.66	

*AB*; arterial blood radioactivity used as input, *P*; unbound plasma radioactivity fraction used as input, *FP*; unbound plasma radioactivity assayed for parent-radiopharmaceutical purity (free parent) used as input. Unpaired two-tailed student's t-test comparing distribution volume of tumors *versus* healthy for each AB, P and FP yielded p values of 1.6×10^-6^, 5.7×10^-6^, and 5.1×10^-4^.

### *Ex Vivo* Histology

The shell-like uptake distribution of the radiopharmaceuticals present in the [^18^F]FDG PET images was seen in autoradiography images confirming tumor uptake distribution pattern. Quantifying sets of brain slice autoradiographs showed that mean uptake ratios of tumor to healthy brain were as follows:

*Ex vivo* histology suggested that central tumor regions were not necrotic in any of the subjects (n = 16, **Table 3**) examined suggesting that it was not the cause of the tumor uptake distribution abnormalities (see **Supplementary Figure 12** for sample) Biodistribution data presented in **Supplementary Figure 13**.

**Table 3:** Autoradiography analysis result summary.

Radiopharmaceutical	Tumor-to-healthy brain uptake ratio	n (slices)
[^18^F]FGln	4.07 ± 0.49	143
[^11^C]Met	1.14 ± 0.41	47
[^18^F]FDG	2.93 ± 0.57	30

## Discussion

The importance of testing new radiopharmaceuticals by contrasting them directly to currently employed standards remains essential in driving the implementation of these new compounds in clinical practice. [^18^F]FGln was tested in orthotopic glioma-bearing BDIX rat subjects successfully and was demonstrated to show improved imaging characteristics *versus* that of [^18^F]FDG and [^11^C]Met. Imaging with all three radiopharmaceuticals was performed on some of the subjects allowing for a direct comparison while reducing total genetic and inter-subject variability. In addition to the multiple radiopharmaceuticals investigated, the two small animal PET/CT camera systems (Molecubes x-cube/β-cube and Inveon Multimodality PET/CT), were used which allowed us to compare their imaging abilities. It was found that, although subject chamber size, image characteristics, and maximum total radioactivity injected are quite different, the normalized end-result data from subjects in both camera groups did not differ significantly.

Qualitative and quantitative comparisons solely via PET/CT image analysis can be inherently biased when delineating the tumor boundaries based upon PET image signals alone. To ensure a non-biased approach to establishing tumor regions, Dotarem^®^ contrast-enhanced MRI images of each subject were taken on the day preceding the first PET/CT images, aligned with each PET/CT, and used to establish tumor boundaries for each individual subject. To prevent "cherry picking" a lower-uptake healthy brain area or inadvertently selecting a healthy area with notably lower uptake for a single radiopharmaceutical, the entire brain was made into a temporary region before an expanded tumor boundary (2 voxels in all x, y and z directions) was subtracted. This whole brain area (minus the expanded tumor region) was then designated as the "healthy brain" and also used for analyses with all three radiopharmaceutical PET images. Having multiple differing-radiopharmaceutical scans of the same subjects not only allowed for a more direct comparison, but also reduced the total number of animals needed to make a substantial comparison by decreasing the random fluctuations in intra-subject differences.

The choice to utilize both of our camera systems served two purposes. With this configuration, we were able to image more animals at a time with a single batch of synthesized radiopharmaceutical while also investigating whether the final results would differ between systems. Comparing the two small PET/CT camera systems directly with *in vivo* subjects, however, can be a bit obfuscated by the inherent variability in individuals despite relative genetic (mouse strain and tumor strain) uniformity. Both camera systems also have many differences in their physical construction as well as technical data output specifics whose overall effect on a study can be difficult to quantify. Physical limitations such as differences in device size, gantry size, imaging volume dimensions, maximum injected radioactivity before sensors are overloaded, and much more must be carefully considered when carrying out and conducting *in vivo* experiments. Admittedly, it is much easier to compare cameras directly by using "phantom subjects" which are inert capsules containing precisely-located chambers that can be filled with known volumes and amounts of radioactivity. While we acknowledge this, we felt it equally important to examine the systems in realistic-use scenarios and propose practically relevant usefulness in comparing end-result data, *e.g.*: normalized ROI values and tumor uptake to healthy brain uptake ratios quantitatively. Our results suggested that, despite camera differences, both *in vivo* image sets are comparable in terms of result-data quality. Using solely either camera would have led to analogous findings and identical conclusions. Bearing in mind that this study is focused primarily on rat brains with the use of heart left ventricle voxels for blood radioactivity estimation, it remains to be seen if imaging with other radionuclides, animal models and *foci* are as comparable for each camera.

The visual comparison of PET cameras from still PET-image slices is difficult and much more can be learned from digitally examining multiple slices and altering image viewing parameters within dedicated software. Many factors were taken into account to contrast the subtle visual differences and a more comprehensive discussion of these parameters is given in the **Supplementary Material** along with additional sample images of common tissues of interest for other studies (*i.e.* [^18^F]FDG heart and brain comparisons and [^18^F]FGln kidney images). The Molecubes' (0.4 × 0.4 × 0.4 mm) voxel size in comparison with the Inveon's (0.78 × 0.78 × 0.80 mm) apparent in the **Supplemental Images**, however, does not entirely quantify their image quality and spatial resolutions. The NEMA spatial resolution of the Inveon system is reported as 1.8 mm (28) whereas the Molecubes system has been reported to be 1.1 mm (18). Both of these are well below the positron range of ^18^F (2.4 mm) and ^11^C (4.2 mm) and in comparison of the size of the ROIs used in this study; we don't expect the resolution differences will play a major role in comparison of the systems. A phantom study comparing these two systems notes that both the Inveon and Molecubes have quite similar qualities in terms of contrast recovery and image quality (19).

Admittedly, the [^11^C]Met scans were mostly unsuccessful for imaging BT4C tumors for unknown reasons as evident from the lack of additional uptake present in PET images. Some autoradiography images showed minimal increase in uptake in the tumor area, though this is a much more sensitive method of *ex vivo* measuring radiopharmaceutical uptake. There was some slightly apparent increased uptake in the extremities of the tumor boundaries in [^11^C]Met PET images (much like the contrast enhanced MRI which functions on the premise of blood brain barrier disruption) though the increase was not present in all subjects and comparably quite minimal. Interestingly, [^11^C]Met uptake in expected tissues such as the salivary glands (**Supplementary Figure 11**) and pancreas (data not shown) was highly present suggesting that there could be an issue with this particular tumor model. One outlier subject with an underdeveloped tumor roughly one third of the size of the rest showed the most tumor uptake relative to the healthy brain, which suggests that an issue may lay with this tumor or animal. Regardless, we felt it beneficial to include this data for future reference as intracranial tumor PET imaging studies with rodents using [^11^C]Met are lacking. Despite reported successes with non-orthotopic brain tumor *loci* (29–32) and with longer physical half-life methionine radiopharmaceuticals such as: [^3^H]Methyl-L-methionine (*in vitro* only) (33) and [^14^C]Methyl-L-methionine (34), it remains to be seen if a successful *in vivo* study using a rodent orthotopic glioma model imaged with [^11^C]Met will be published.

[^18^F]FDG imaging performed was largely successful, though it was observed that peak radiopharmaceutical uptake might not have had enough time to occur. We did not explore longer additional imaging sessions due to practical reasons. Although [^18^F]FDG uptake was in most cases also noticeably higher in extremities of the tumor boundary, the *ex vivo* studies refuted the notion that central tumor necrosis was present in any of the subjects. The shell-like distribution was also confirmed to not be a PET image reconstruction artifact when examining the *ex vivo* autoradiography which mimicked the observed uptake shell (**Supplementary Figure 12**).

[^18^F]FGln imaging was successful and suggested an improved ability to PET image gliomas *in vivo* evident by the increased tumor to healthy brain uptake ratio. The modeling studies reinforced previous findings (22), as well as agreed with general literature understanding of glutamine uptake biochemistry, that reversible uptake plays the most significant role in pharmacokinetics. The rapid metabolism rate in rats (approximately two fold that of mice) made it clear that correcting the blood radioactivity inputs for intact [^18^F]FGln had an even more obvious impact on modeling results as, after one hour of radiopharmaceutical injection, the actual amount of free intact [^18^F]FGln in the blood was only 28% of the total radioactivity present. Despite suggestions that correcting the blood inputs may not have much of an impact due to the metabolism occurring primarily elsewhere (35), we propose that modeling based on blood radioactivity level alone when roughly 70% of the input value is either bound to proteins or a different molecule entirely may not be the most accurate approach. One issue though, however, is that the other metabolites [primarily (2*S*,4*R*)-4-[^18^F]fluoroglutamate (24)] have their own pharmacokinetics making modeling of the system a very complex endeavor. Due to this, we have provided data tables for multiple models in the **Supplementary Material** for corrected and uncorrected blood inputs with the hope that data may find use for future comparisons- whether different animal strains or radiopharmaceutical modifications.

There will not always be a single preferential imaging radiopharmaceutical due to the unique cell biology and biochemistry present in different cancer strains. It remains important to contrast and assess emerging imaging compounds for specific cancer types and push forward the era of personalized medicine. Comparing and quantifying new radiopharmaceuticals for potential improvements to PET imaging is essential for the continued progress of the field and we hope that this work will lead to more comparative *in vivo* studies with different cameras, radiopharmaceuticals, and pharmacokinetic models.

In this study, we present improved *in vivo* orthotopic-glioma PET imaging characteristics of [^18^F]FGln *versus* [^18^F]FDG and [^11^C]Met and a rich collection of pharmacokinetic [^18^F]FGln models in BDIX rats. We also report that for the radiopharmaceuticals and *foci* investigated, both PET/CT imaging systems (the Molecubes and Inveon PET/CT) provided no statistically significant differences in end-result *in vivo* imaging data.

## Data Availability Statement

The raw data supporting the conclusions of this article will be made available by the authors, without undue reservation.

## Ethics Statement

The animal study was reviewed and approved by The National Animal Experiment Board of Finland and the Regional State Administrative Agency for Southern Finland.

## Author Contributions

MM, HL, JV, and PE carried out many of the experiments. SH and OE synthesized and analyzed batches of [^11^C]Met and [^18^F]FDG. JT and KS developed and implemented MRI scanning. VO conducted modeling analyses. GY developed methods for and synthesized the [^18^F]FGln precursor. AK-R bred and provided the breeding pair of BDIX rats for the animal model. HM, X-GL, and AR supervised, reviewed, and corrected the manuscript. All authors contributed to the article and approved the submitted version.

## Funding

The study was financially supported by grants from the State Research Funding of Turku University Hospital, the Jane and Aatos Erkko Foundation, the Finnish Cultural Foundation, and the Drug Research Doctoral Programme of the University of Turku Graduate School. X-GL give thanks for financial support from the InFlames Flagship Programme of the Academy of Finland (decision number 337530).

## Conflict of Interest

The authors declare that the research was conducted in the absence of any commercial or financial relationships that could be construed as a potential conflict of interest.

## Publisher’s Note

All claims expressed in this article are solely those of the authors and do not necessarily represent those of their affiliated organizations, or those of the publisher, the editors and the reviewers. Any product that may be evaluated in this article, or claim that may be made by its manufacturer, is not guaranteed or endorsed by the publisher.
