# Supplementary material for: Comparison of: (2S,4R)-4-[18F]Fluoroglutamine, [11C]Methionine, and 2-Deoxy-2-[18F]Fluoro-D-Glucose and Two Small-Animal PET/CT Systems Imaging Rat Gliomas
Source: Front Oncol. 2021 Oct 7;11:730358. doi: 10.3389/fonc.2021.730358 (PMC8530378; doi:10.3389/fonc.2021.730358)
Supplement: Supplementary file 1 [file DataSheet_1.docx]

***Supplementary Material***

**Comparison of: (2*S*,4*R*)-4-[^18^F]Fluoroglutamine, [^11^C]Methionine, and 2-deoxy-2-[^18^F]fluoro-*D*-glucose and Two Small-Animal PET/CT Systems Imaging Rat Gliomas**

**Maxwell WG Miner^1^, Heidi Liljenbäck^1,2^, Jenni Virta^1^, Semi Helin^1^, Olli Eskola^1^, Petri Elo^1^, Jarmo Teuho^3^, Kerttu Seppälä^3^, Vesa Oikonen^1^, Guangli Yang^4^, Andrea Kindler-Röhrborn^5^, Heikki Minn^1,6^, Xiang-Guo Li^1,7^, Anne Roivainen^1,2,3,7^**

*^1^Turku PET Centre, University of Turku, FI-20520 Turku, Finland*

*^2^Turku Center for Disease Modeling, University of Turku, FI-20014 Turku, Finland*

*^3^Turku PET Centre, Turku University Hospital, FI-20520 Turku, Finland*

*^4^Memorial Sloan Kettering Cancer Center, Organic Synthesis Core Facility, 1275 York Ave. NY, USA*

*^5^Institute of Pathology, University Hospital of Essen, University of Duisburg-Essen, DE-45147 Essen, Germany*

*^6^Department of Oncology and Radiotherapy, Turku University Hospital, FI-20520 Turku, Finland*

*^7^InFLAMES Research Flagship Center, University of Turku, FI-20520, Turku, Finland*

**Correspondence:**Anne Roivainen
Anne.Roivainen@utu.fi

**Additional Methods and Results**

*[^11^C]Methionine Production*

The *L*-[methyl-^11^C]methionine ([^11^C]Met) was prepared using a TracerLab FXC-Pro synthesis module equipped with AgOTf/graphitized carbon column for on-line conversion of produced [^11^C]methyl iodide into [^11^C]methyl triflate.

0.1 M sodium phosphate solution was obtained from Turku University Hospital, Hospital Pharmacy. Ultra-pure water was produced on-site. All other chemicals were acquired from commercial suppliers.

[^11^C]Methyl triflate was bubbled into a solution of L-homocysteine (1.0 mg), 0.5 M sodium hydroxide (15 µl) and ultra-pure water (300 µl) at 1 °C, followed by heating the reaction solution to 60 °C for 1 minute. HPLC mobile phase (10 mM NaH_2_PO_4_, 0.5 ml) was added before injection into Phenomenex Gemini® C18 (250 × 10 mm, 10 µm) HPLC column. Separation was monitored with detectors for radioactivity and UV absorbance at 225 nm. Product fraction was collected into a vessel containing 0.1 M sodium phosphate solution (5.5 ml) and ethanol (0.5 ml). Finally the solution was filtered through a Merck Millipore Millex GV 0.22 µm sterile filter.

Waters µBondapak NH_2_, 300 × 3.9 mm, 10 µm HPLC column was used for radiochemical purity and chemical purity analysis, and Daicel Crownpak CR(+) column at 7 °C for optical purity determination. (A) 10 mM KH_2_PO_4_, pH 4.3 and (B) CH_3_CN/H_2_O, 50/7 at 2 ml/min were used for radiochemical purity and identity in a gradient run; 5% A at 0 min, 50 % B at 10 min, 50 % B at 14 min, 5% B at 16 min. Isocratic 0.6 ml/min flow of 20 mM HClO_4_ was used for optical purity.

*Unbiased MRI-guided PET image analyses*


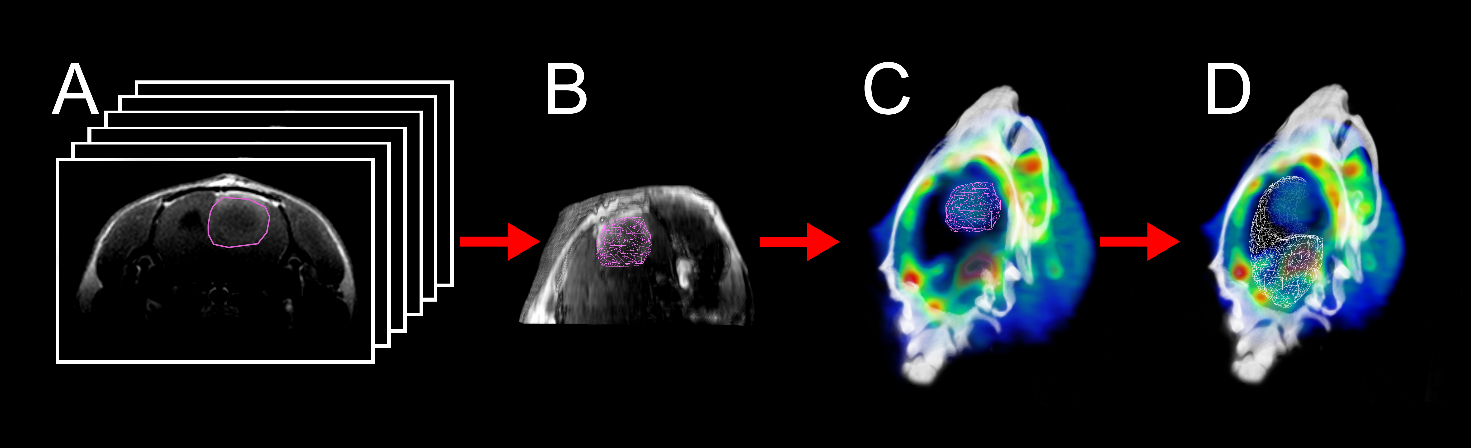

**SUPPLEMENTARY FIGURE 1.** Unbiased analysis method workflow. (**A**) Tumor boundaries established through transaxial sections of MRI. (**B**) Boundaries are converted into 3D space. (**C**) MRI with 3D ROI is aligned with all PET Images. (**D**) Healthy brain region established by subtracting an expanded tumor volume from a whole-brain ROI. *note the 3-D nature and depth of field in the images makes it appear that high activity areas are selected in the healthy brain mesh in part “**D**” even though they are behind the mesh itself.

**Additional Results**

*Visual Camera Comparisons*


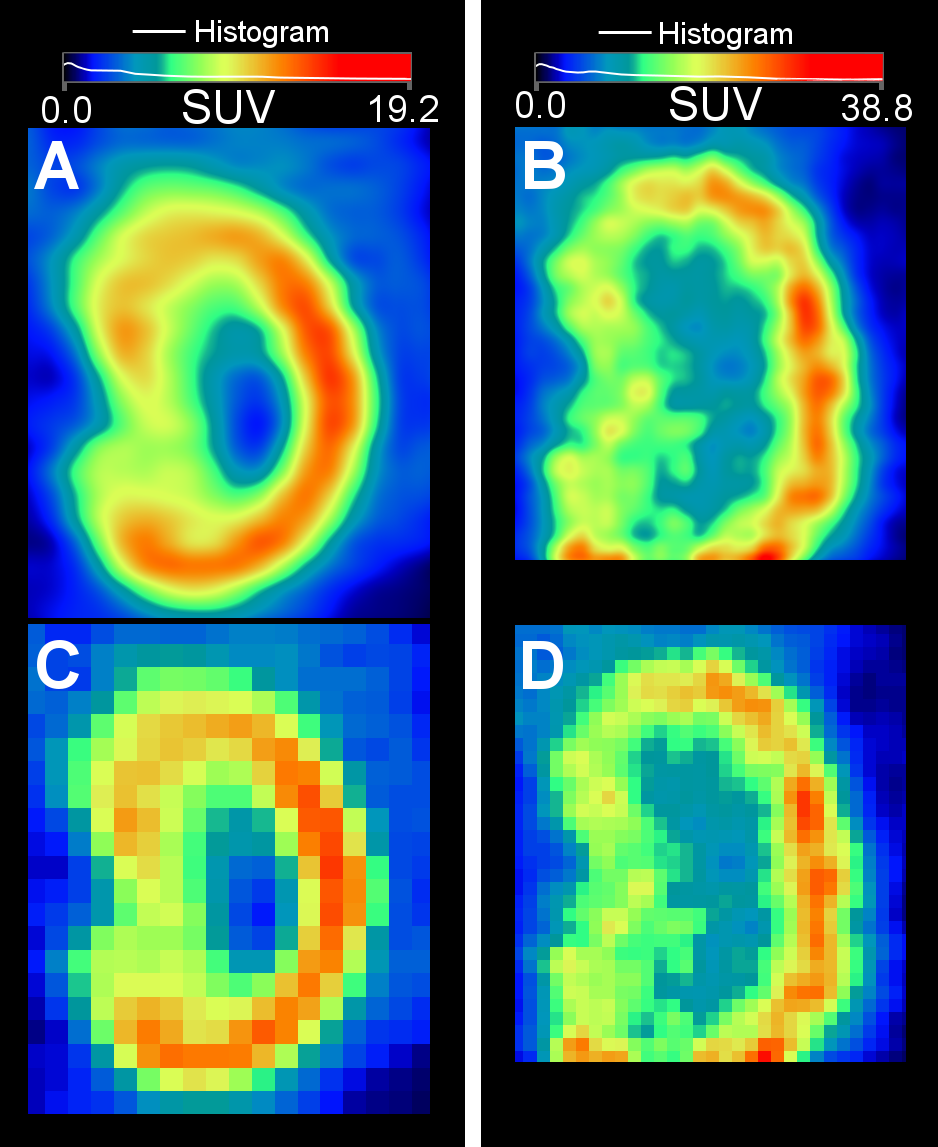


**SUPPLEMENTARY FIGURE 2.** Visual comparison of coronal [^18^F]FGln PET image slice of a BDIX rat kidney time weighted means from 4-40 min post-injection. (**A**) and (**C**) 15.2 MBq injected subject imaged with the Inveon Multimodality PET/CT camera (tri-cubic and nearest-neighbour interpolations respectively). (**B**) and (**D**) 4.2 MBq injected subject imaged with the Molecubes β-cube (tri-cubic and nearest-neighbour interpolations, respectively).


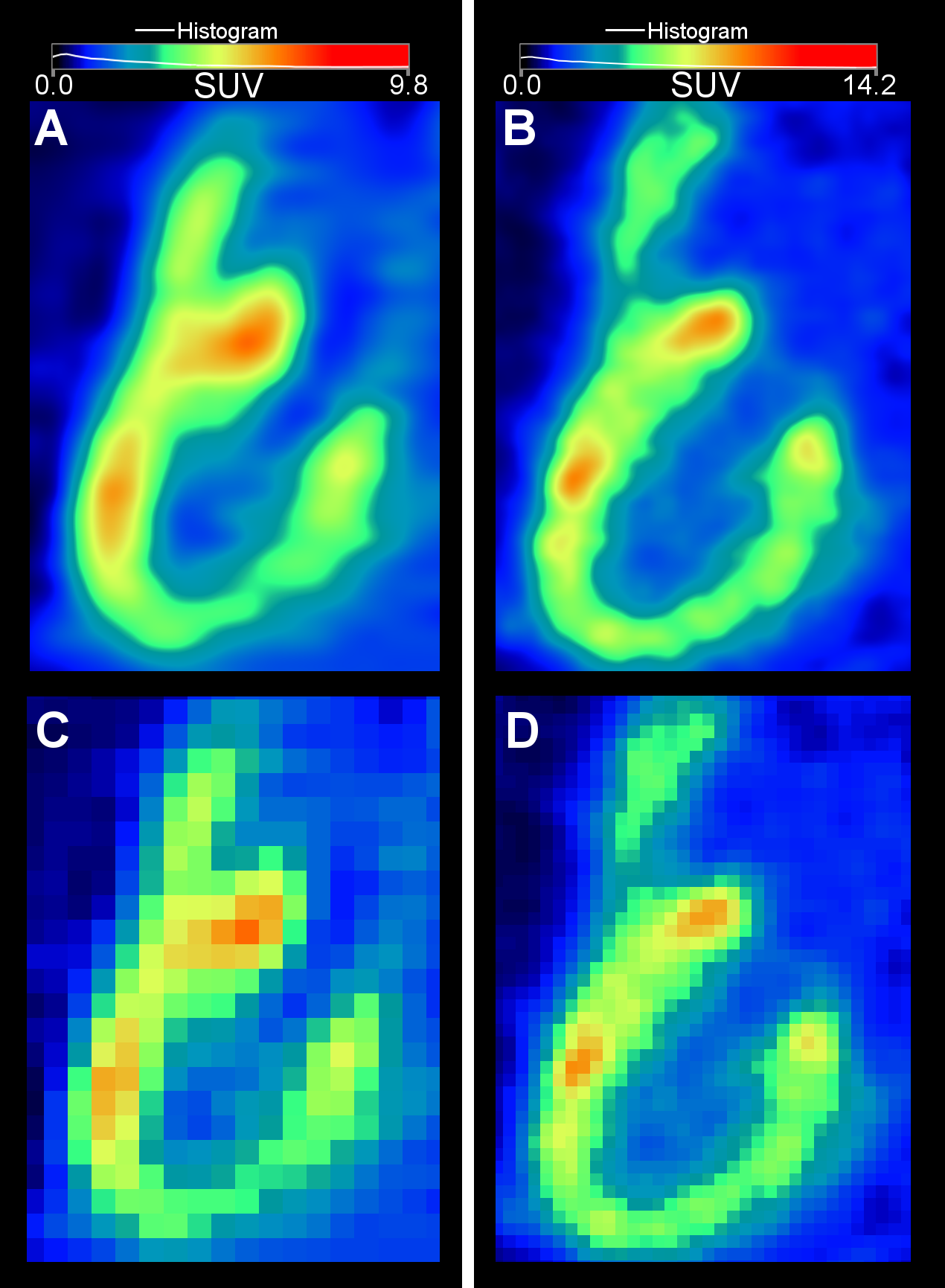


**SUPPLEMENTARY FIGURE 3.** Visual comparison of coronal [^18^F]FDG PET image slice of a BDIX rat heart time-weighted means from 20-60 min post-injection. (**A**) and (**C**) 23.7 MBq injected subject imaged with the Inveon Multimodality PET/CT camera (tri-cubic and nearest-neighbor interpolations respectively). (**B**) and (**D**) 7.8 MBq injected subject imaged with the Molecubes β-cube (tri-cubic and nearest-neighbor interpolations, respectively).


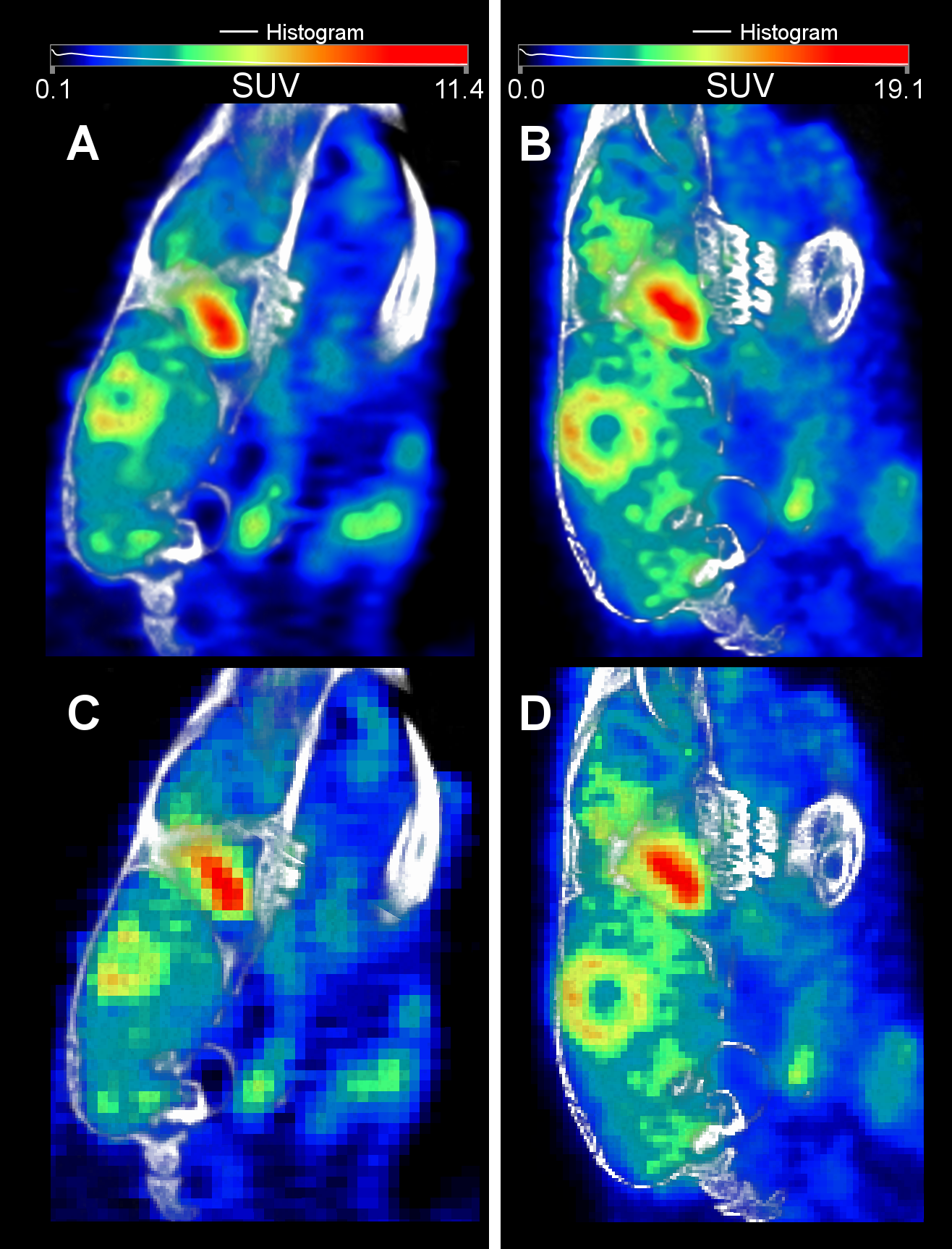


**SUPPLEMENTARY FIGURE 4.** Visual comparison of sagittal [^18^F]FDG PET/CT image slice of a BDIX rat head and neck area of time-weighted means from 20-60 min post-injection. (**A**) and (**C**) 23.7 MBq injected subject imaged with the Inveon Multimodality PET/CT camera (tri-cubic and nearest-neighbor interpolations respectively). (**B**) and (**D**) 7.8 MBq injected subject imaged with the Molecubes X-cube/β-cube (tri-cubic and nearest-neighbor interpolations, respectively).

*In Vivo Stability*

*
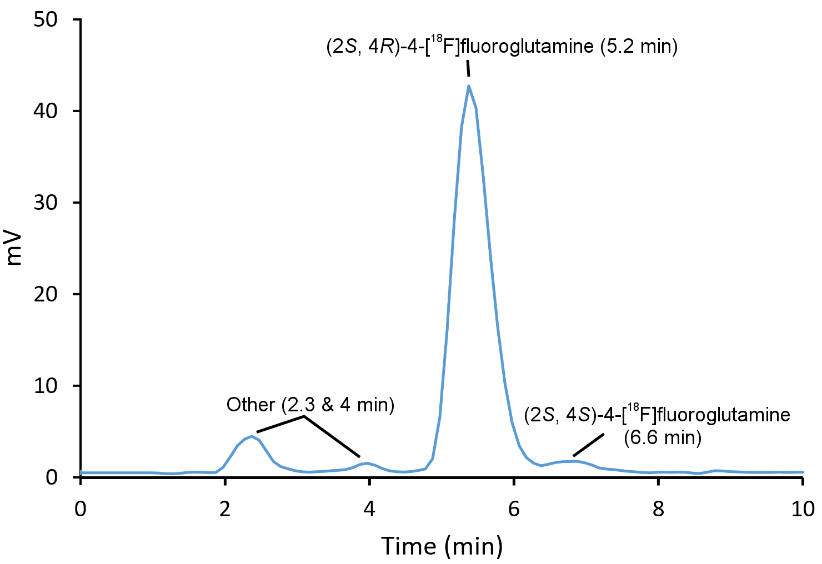
*

**SUPPLEMENTARY FIGURE 5.** Sample stability HPLC flow scintillation chromatogram for (relative) [^18^F]FGln plasma precipitated purity assay.


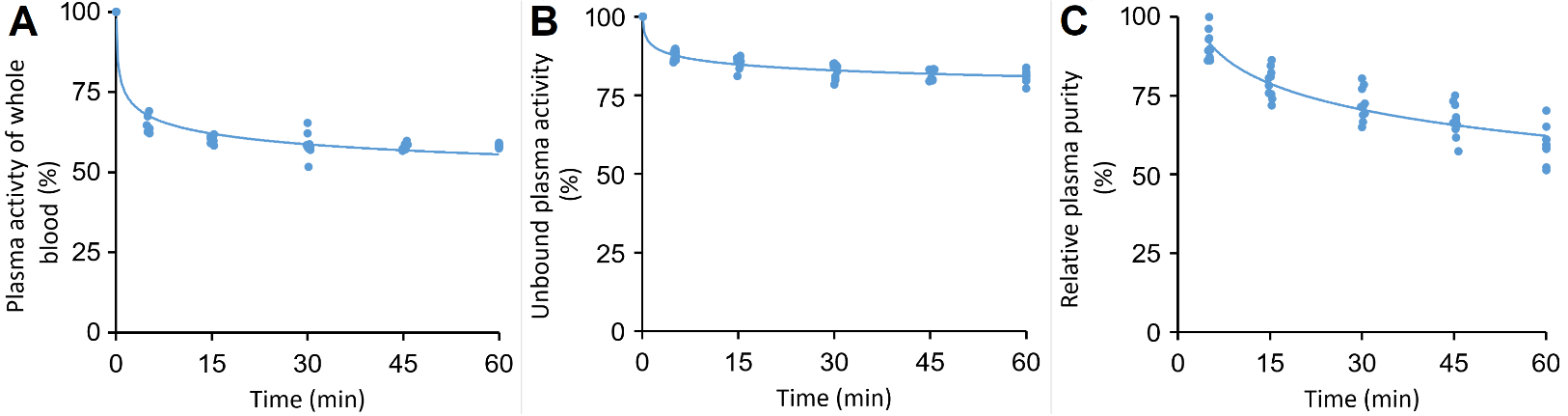


**SUPPLEMENTARY FIGURE 6.** *In vivo* stability analyses assay data accounting for fractions of [^18^F]FGln unavailability. (**A**) Plasma radioactivity fraction of whole blood (100% ­ erythrocyte uptake %) with fitted curve: *y* = 0.7694*x*^-0.08^, R^2^ = 0.950. (**B**) Unbound radioactivity fraction of methanol-precipitated plasma with fitted curve: *y* = -0.027ln(*x*) + 0.9208, R^2^ = 0.948. (**C**) Relative (adjusted for injected radiopharmaceutical purity) purity of the unbound precipitated plasma samples from “**B**” assayed via HPLC with fitted curve: *y* = -0.12ln(*x*) + 1.1118, R^2^ = 0.816.


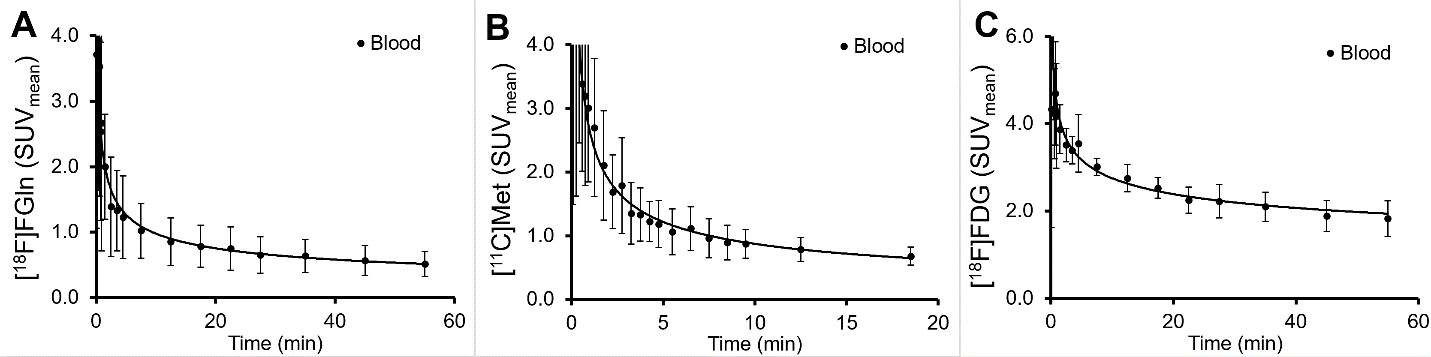


**SUPPLEMENTARY FIGURE 7.** Average PET image-derived time-radioactivity curves of all subjects’ PET/CT image blood ROI’s (**A**) [^18^F]FGln (n = 13), (**B**) [^11^C]Met (n = 12), (**C**) [^18^F]FDG (n = 7).

*Modeling*


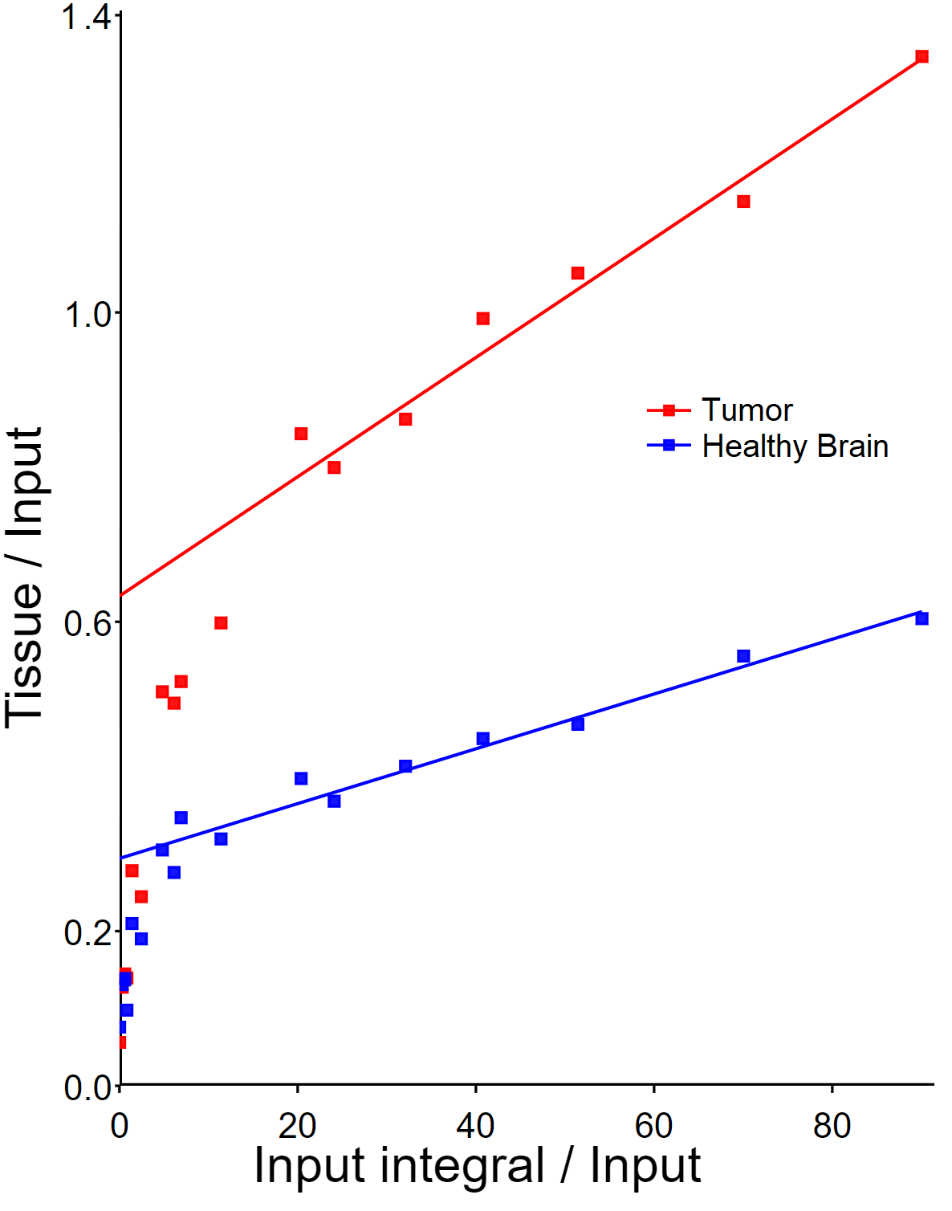


**SUPPLEMENTARY FIGURE 8.** Sample [^18^F]FGln Patlak plot with arterial blood used as the input.

**SUPPLEMENTARY TABLE 1.** Patlak plot (irreversible uptake model) [^18^F]FGln result summary for arterial blood, unbound plasma, and metabolite corrected unbound plasma fraction of arterial blood inputs

| **Region of interest** | **K_i_ (AB)** | **Ic (AB)** | **r (AB)** | **K_i_ (P)** | **Ic (P)** | **r (P)** | **K_i_ (FP)** | **Ic (FP)** | **r (FP)** |
| --- | --- | --- | --- | --- | --- | --- | --- | --- | --- |
| Tumor  (n = 13) | 0.011  ± 0.004 | 0.676  ± 0.221 | 0.962  ± 0.066 | 0.008  ± 0.003 | 0.418  ± 0.149 | 0.974  ± 0.042 | 0.023  ± 0.006 | 0.823  ± 0.376 | 0.993  ± 0.009 |
| Healthy Brain  (n = 13) | 0.005  ± 0.003 | 0.376  ± 0.114 | 0.949  ± 0.093 | 0.004  ± 0.002 | 0.236  ± 0.076 | 0.965  ± 0.061 | 0.011  ± 0.004 | 0.495  ± 0.195 | 0.990  ± 0.018 |

*AB*; arterial blood radioactivity used as input, *P*; unbound plasma radioactivity fraction used as input, *FP*; unbound plasma radioactivity assayed for parent tracer purity (free parent) used as input. *K_i_* = net influx rate (min^-1^), *Ic* = y intercept.


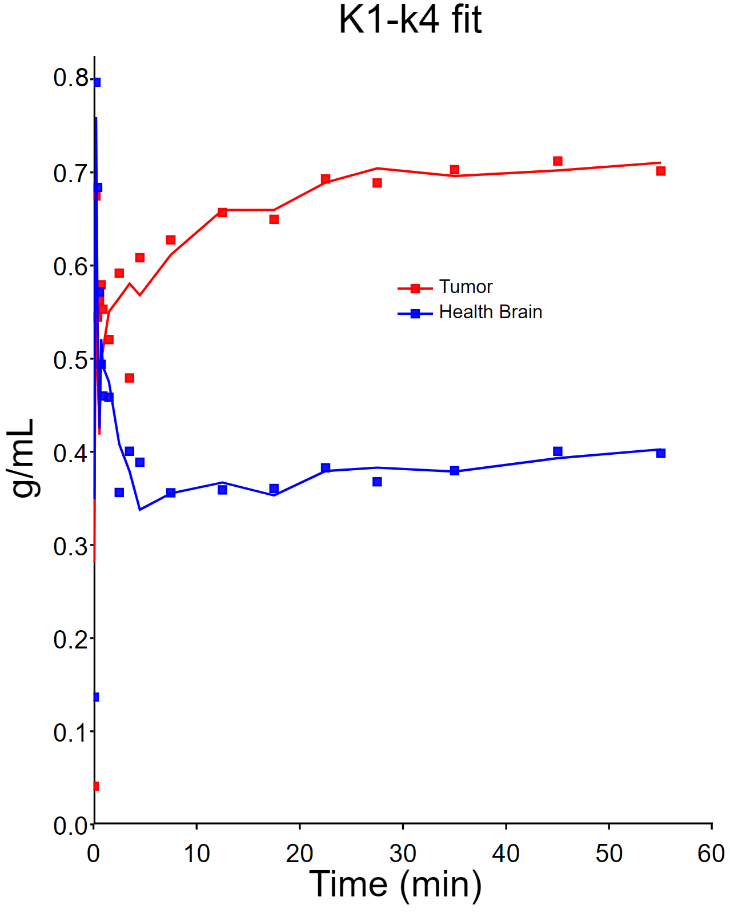


**SUPPLEMENTARY FIGURE 9.** Sample [^18^F]FGln reversible two-tissue compartment model.


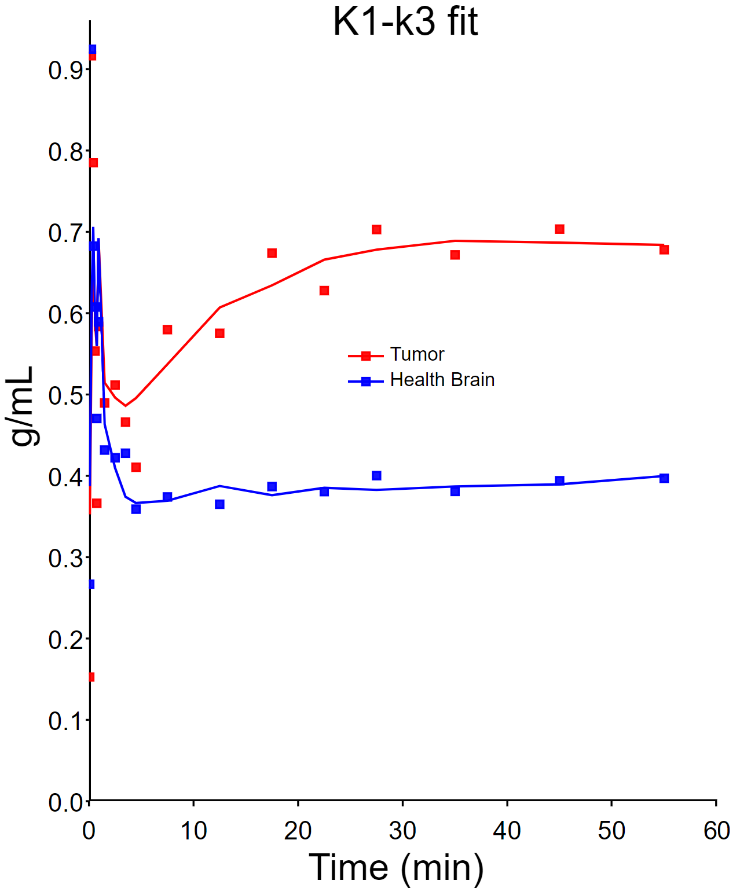


**SUPPLEMENTARY FIGURE 10.** Sample [^18^F]FGln irreversible two-tissue compartment model.

**SUPPLEMENTARY TABLE 2.** Reversible two-tissue compartment-model [^18^F]FGln result summary for arterial blood, unbound plasma, and metabolite corrected unbound plasma fraction of arterial blood inputs

| **Region of interest** | **K_1_**  **(AB)** | **K_1_/k_2_ (AB)** | **k_3_**  **(AB)** | **k_3_/k_4_ (AB)** | **K_1_**  **(P)** | **K_1_/k_2_**  **(P)** | **k_3_**  **(P)** | **k_3_/k_4_**  **(P)** | **K_1_**  **(FP)** | **K_1_/k_2_ (FP)** | **k_3_**  **(FP)** | **k_3_/k_4_ (FP)** |
| --- | --- | --- | --- | --- | --- | --- | --- | --- | --- | --- | --- | --- |
| **Tumor**  **(n =13)** | 0.133  ± 0.079 | 0.551  ± 0.221 | 0.073  ± 0.054 | 2.901  ± 1.331 | 0.052  ± 0.026 | 0.331  ±0.125 | 0.231  ± 0.478 | 3.191  ± 1.559 | 0.057  ± 0.023 | 1.646  ± 2.403 | 0.291  ± 0.481 | 4.466  ± 1.378 |
| **Healthy Brain**  **(n = 13)** | 0.118  ± 0.132 | 0.248  ± 0.128 | 0.060  ± 0.039 | 3.279  ± 1.395 | 0.041  ± 0.031 | 0.137  ± 0.062 | 0.086  ± 0.039 | 4.021  ± 1.249 | 0.033  ± 0.023 | 0.528  ± 0.363 | 0.196  ± 0.354 | 4.462  ± 1.312 |

*AB*; arterial blood radioactivity used as input, *P*; unbound plasma radioactivity fraction used as input, *FP*; unbound plasma radioactivity assayed for parent tracer purity (free parent) used as input. *K_1_* = plasma to tissue transport rate or unidirectional transport rate across blood brain barrier (mL min^-1^ mL^-1^), *K_1_*/*k_2_* = equilibrium distribution volume of radioligand in the tumor (mL mL^-1^), *k_3_* = intracellular to intercellular rate constant (min ^-1^), *k_4_* = intercellular to intracellular rate constant (min ^-1^), *k_3_/k_4_* = distribution volume between intracellular and intercellular compartments (min min^-1^).

**SUPPLEMENTARY TABLE 3.** Irreversible two-tissue compartment-model [^18^F]FGln result summary for arterial blood, unbound plasma, and metabolite corrected unbound plasma fraction of arterial blood inputs

| **Region of interest** | **K_1_**  **(AB)** | **K_1_/k_2_ (AB)** | **k_3_**  **(AB)** | **K_i_**  **(AB)** | **k_3_/(k_2_+k_3_)**  **(AB)** | **K_1_**  **(P)** | **K_1_/k_2_**  **(P)** | **k_3_**  **(P)** | **K_i_**  **(P)** | **k_3_/(k_2_+k_3_)**  **(P)** |
| --- | --- | --- | --- | --- | --- | --- | --- | --- | --- | --- |
| **Tumor**  **(n = 13)** | 0.088  ± 0.039 | 0.876  ± 0.29 | 0.017  ± 0.005 | 0.012  ± 0.004 | 0.156  ± 0.078 | 0.037  ± 0.015 | 0.685  ± 0.330 | 0.017  ± 0.007 | 0.008  ± 0.003 | 0.226  ± 0.078 |
| **Healthy Brain**  **(n = 13)** | 0.063  ± 0.053 | 0.338  ± 0.159 | 0.022  ± 0.010 | 0.006  ± 0.002 | 0.126  ± 0.068 | 0.024  ± 0.017 | 0.221  ± 0.105 | 0.028  ± 0.014 | 0.004  ± 0.001 | 0.215  ± 0.096 |

*AB*; arterial blood radioactivity used as input, *P*; unbound plasma radioactivity fraction used as input. *K_1_* = plasma to tissue transport rate or unidirectional transport rate across blood brain barrier (mL min^-1^ mL^-1^), *K_1_*/*k_2_* = equilibrium distribution volume of radioligand in the tumor (mL mL^-1^), *k_3_* = intracellular to intercellular rate constant (min ^-1^), *K_i_* = Net influx rate constant (min ^-1^)**,** k_3_/(k_2_+k_3_) **=** approximation fraction or distribution volume of transported ligand from tumor out to either further metabolism or healthy brain (min min^-1^).

**SUPPLEMENTARY TABLE 4** Continued.

| **Region of interest** | **K_1_**  **(FP)** | **K_1_/k_2_**  **(FP)** | **k_3_**  **(FP)** | **K_i_**  **(FP)** | **k_3_/(k_2_+k_3_)**  **(FP)** |
| --- | --- | --- | --- | --- | --- |
| **Tumor**  **(n = 13)** | 0.083  ± 0.113 | 1.841  ± 0.967 | 0.105  ± 0.246 | 0.023  ± 0.009 | 0.385  ± 0.157 |
| **Healthy Brain**  **(n = 13)** | 0.033  ± 0.018 | 0.524  ± 0.324 | 0.064  ± 0.063 | 0.012  ± 0.003 | 0.420  ± 0.146 |

*FP*; unbound plasma radioactivity assayed for parent tracer purity (free parent) used as input. Variables described above.

**
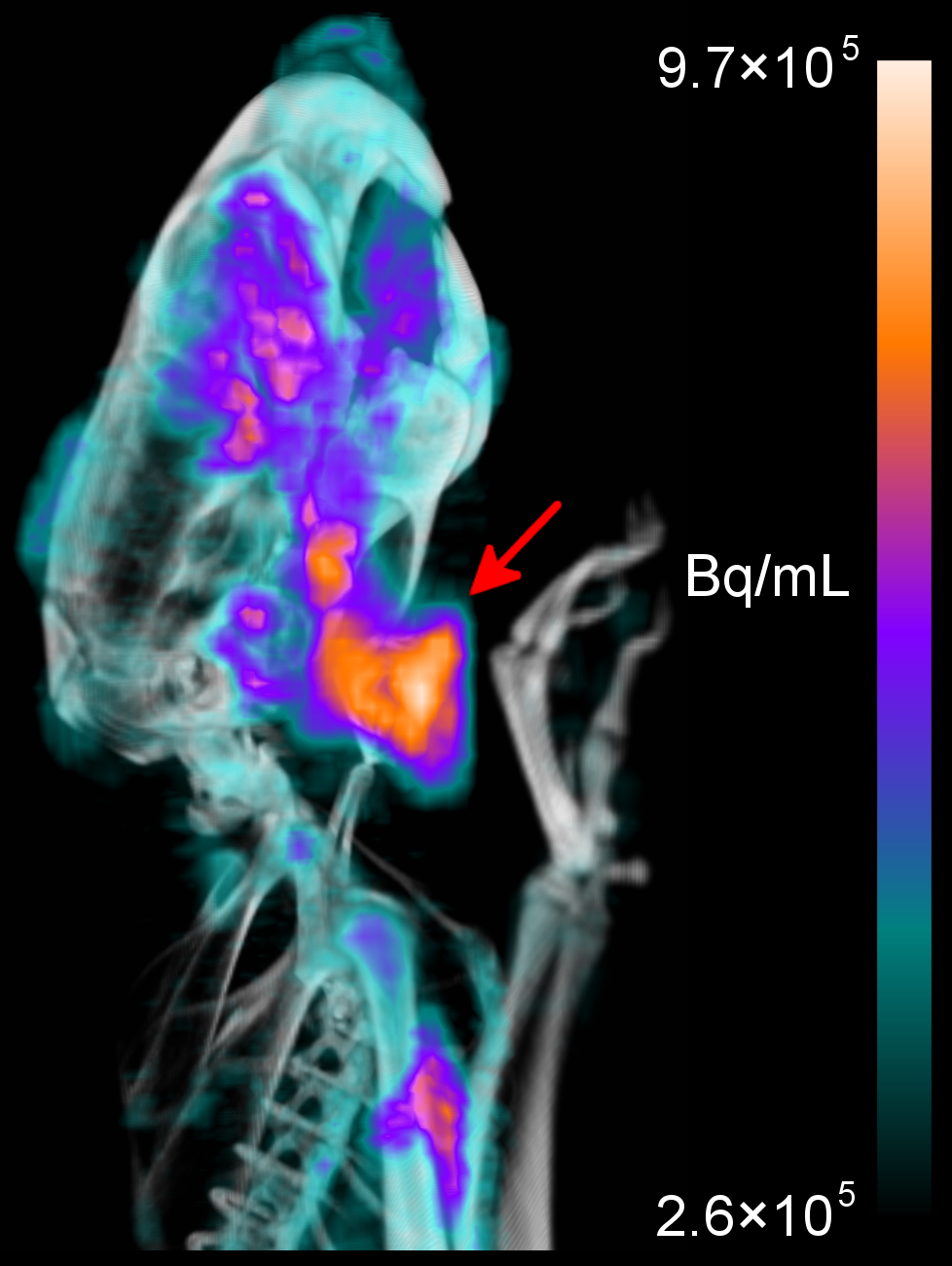
**

**SUPPLEMENTARY FIGURE 11.** *In vivo* [^11^C]Met PET/CT 3D-fusion image (maximum intensity projection time-weighted mean of PET frames from 5-20 min) showing high uptake in the salivary glands (red arrow).

*Ex Vivo Stu ssdies*

The excised brains containing tumors were frozen with liquid nitrogen, sectioned with a Leica CM3050S cryotome into alternating 20 and 8 µm slices on different slides for hematoxylin/eosin and future histochemical staining respectively. The 20 µm slides from each radiopharmaceutical were exposed to autoradiography imaging plates for 2 to 3 × their respective radioisotope half-life and scanned with a Fujifilm BAS-5000 system (Fujifilm, Tokyo, Japan). The slides were then stained with hematoxylin/eosin and digitally scanned with a Pannoramic 250 Flash slide scanner (3DHistech, Budapest, Hungary) Autoradiographs and light micrographs were then aligned in Carimas software (Turku PET Centre, Turku, Finland) and analyzed therein.


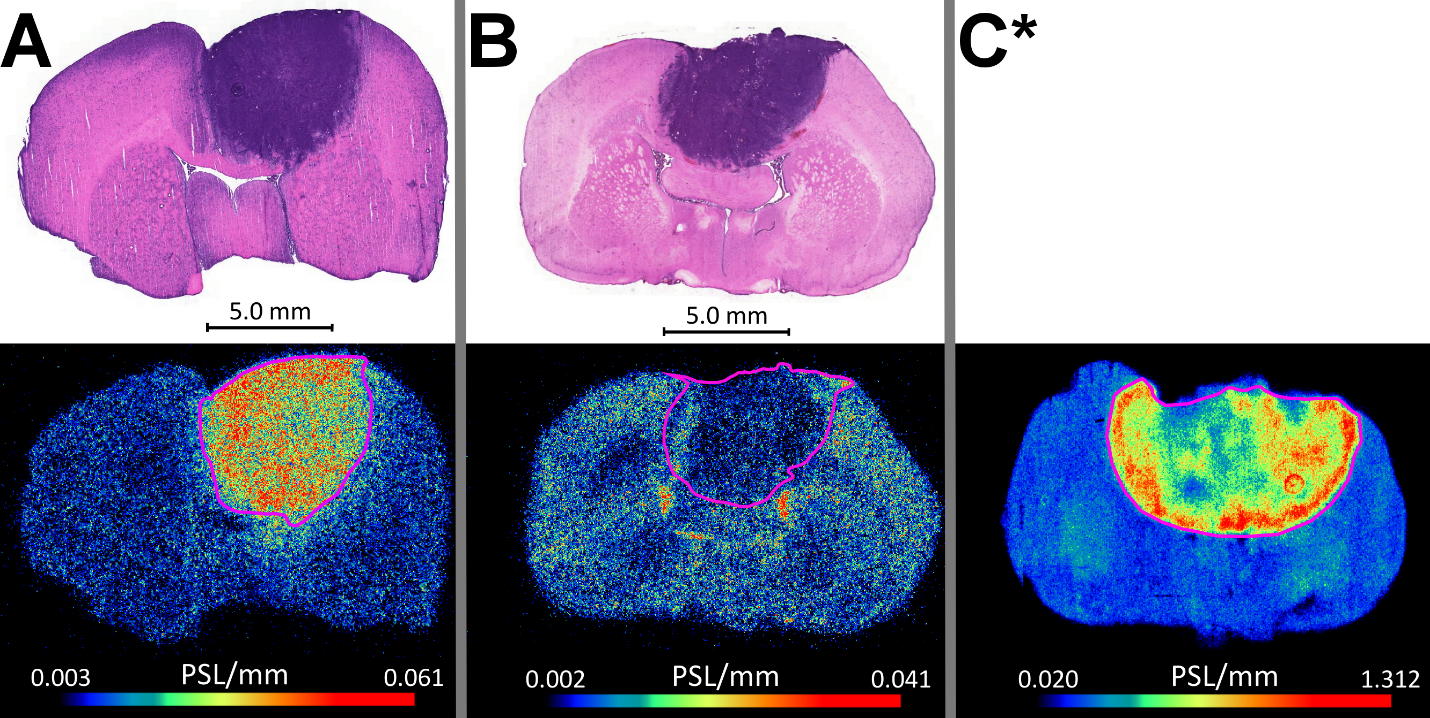


**SUPPLEMENTARY FIGURE 12.** *Ex vivo* histological comparison with upper sections showing Hematoxylin/eosin staining and bottom showing autoradiographs of 20 µm transaxial brain cryosections from BDIX rats with BT4C gliomas (circled in magenta) injected with (**A**) [^18^F]FGln, (**B**) [^11^C]Met, or (**C**) [^18^F]FDG. *note: unfortunately the corresponding cryosections for the [^18^F]FDG samples were lost after scanning the autoradiographs.

*[^18^F]FGln Biodistribution*

Two 60 minute post-[^18^F]FGln-injection subjects along with three subjects after 60 minute dynamic [^18^F]FGln PET imaging were sacrificed for autoradiography (where applicable) and biodistribution studies.The following tissues were dissected, weighed and assayed for radioactivity levels with a 3″ Nal well counter (Triathler, Hidex, Turku, Finland): Cerebellum, healthy brain, olfactory bulb, muscle, heart, lung, liver, spleen, pancreas, kidney, stomach (empty), small intestine (empty), large intestine (empty), lymph node, white adipose tissue (WAT), brown adipose tissue (BAT), bone (skull), femur (bone + marrow), epididymis, and testis, or uterus and ovaries. Radioactivity values were back corrected for decay to injection time, amount of tracer injected and the weight of the subject to calculate standardized uptake values (SUV) and % injected radioactivity dose per tissue weight (g) (%ID/g).


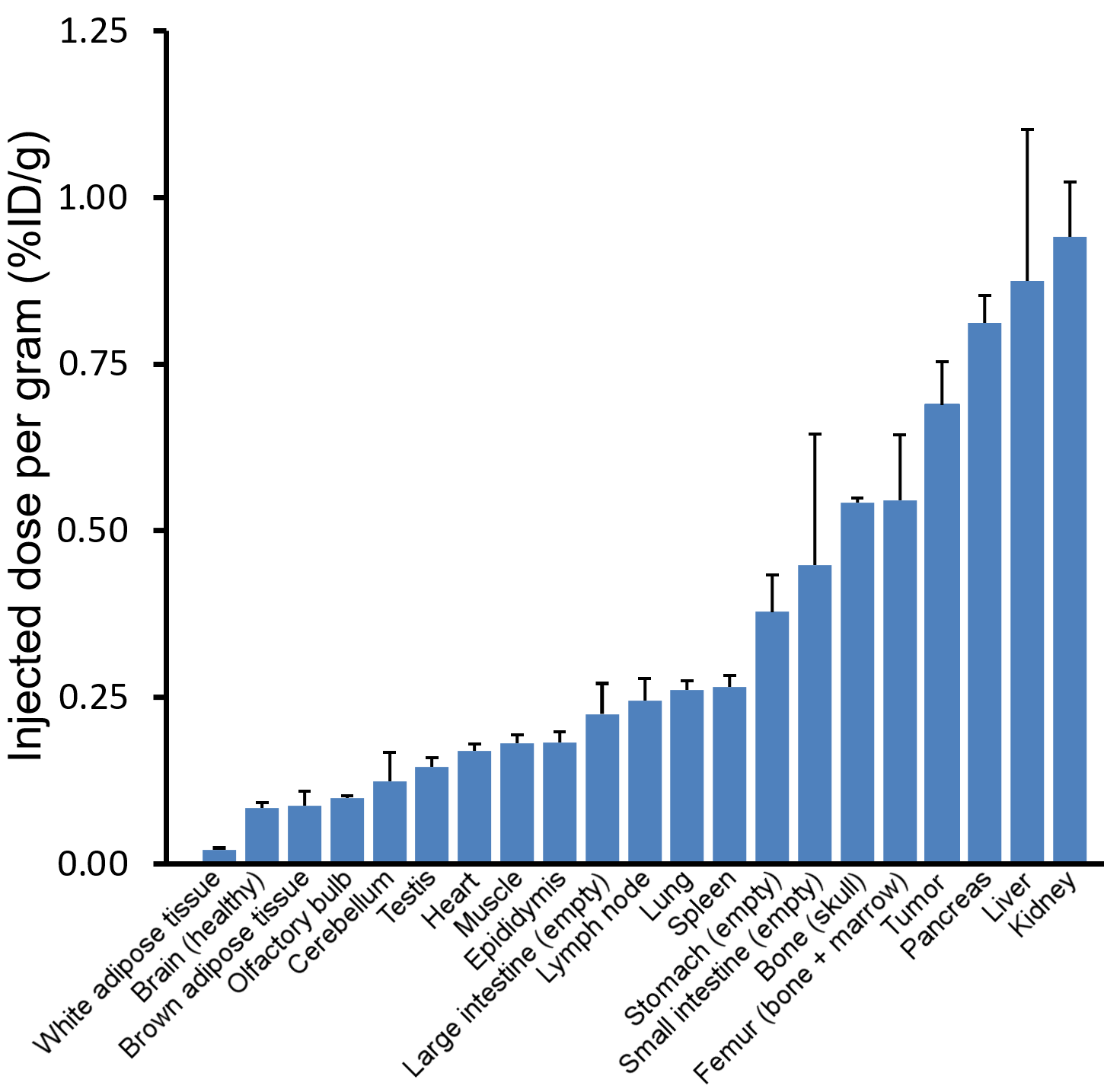


**SUPPLEMENTARY FIGURE 13.** Average (n = 5) biodistribution for BDIX rats injected with 24.5 ± 8.7 MBq [^18^F]FGln.

**Additional Discussion**

*Image Analysis Comparison Approach*

The interpolation method chosen (nearest neighbor, tri-linear, or tri-cubic) can affect visual display and along with smoothing operations in camera processing software can suppress inherent noise. We chose to display tri-cubic as it generally reveals image noise more clearly. We also included nearest-neighbor interpolations to display voxel size differences. The summation operation chosen for the frames (number of frames, simple mean, time-weighted mean, or sum) may favor certain analyses and specific-radiopharmaceutical pharmacokinetics. In this regard, we utilized a time-weighted means of the same periods for both systems, which were selected dependent on target tissue to display each most effectively. Color scale choice, or how the scale is applied to the distribution of voxel values (highly dependent on injected activity and tissue investigated), can also mask or highlight essential information. With respect to this, we created a new color scale to have a larger dynamic range in the middle-region colors to more visually display subtle differences here. The color scale was then applied relatively to the image voxel-distribution histogram instead of mimicking the same SUV range to ensure a better visual match. It is likely that the SUV-scale ranges differ slightly between camera systems due to the large (3-5 times) difference in injected radioactivity between them which can affect the images through many possible pathways.

Admittedly, an impeccable amount of trust must be bestowed upon the skilled image analyst to make such visual comparisons in an unbiased fashion. In this respect, we have carefully prepared and presented images to highlight the minute differences in the PET image data from each of the camera systems. Ultimately, we propose that there were not significant differences in normalized end-result data for blood (heart left ventricle), tumor, or healthy brain tissue for the models and radiopharmaceuticals investigated. Continued investigation into what differences that were observed (Solely the slightly higher TBR for the Inveon system for [^18^F]FGln) is ongoing.

*Modeling Discussion*

The modeling results represent only an approximation and estimation of the reality as the main metabolism product (fluoroglutamate) has its own uptake pharmacokinetics. The results represent an oversimplification of the real pharmacokinetics and are more akin to a combined result of both radiopharmaceuticals. Still we propose some usefulness concerning future developments of the [^18^F]FGln radiopharmaceutical and testing in the BDIX rat strain. The bioavailability corrections in the metabolism study offered a way to correct for unavailable radiopharmaceutical in the blood, but it is possible that correcting for all aspects in combination “over-corrects” the result. When examining the distribution volumes (DV) of the two-compartment model results, it appears that the DV that most closely resembles the tumor and healthy brain SUV from PET images is the plasma-corrected (P) which takes into account the red blood cell uptake unavailability and protein binding without accounting for HPLC purity of the plasma components (*i.e.* fluoroglutamate component). In this regard, it is likely that future metabolism studies could be omitted with this radiopharmaceutical when testing BDIX rats and the plasma correction curve used to approximate availability of “[^18^F]FGln” for modeling purposes, but more studies would be needed to confirm this.
